# Dynamics of SARS-CoV2 Infection and Multi-Drug Resistant Bacteria Superinfection in Patients With Assisted Mechanical Ventilation

**DOI:** 10.3389/fcimb.2021.683409

**Published:** 2021-08-12

**Authors:** Annarita Mazzariol, Anna Benini, Ilaria Unali, Riccardo Nocini, Marcello Smania, Anna Bertoncelli, Francesco De Sanctis, Stefano Ugel, Katia Donadello, Enrico Polati, Davide Gibellini

**Affiliations:** ^1^Microbiology Section, Department of Diagnostics and Public Health, University of Verona, Verona, Italy; ^2^Pharmacology Section, Department of Diagnostics and Public Health, University of Verona, Verona, Italy; ^3^Department of Otolaryngology-Head and Neck Surgery, University Hospital of Verona, Verona, Italy; ^4^Unità Operativa Complessa of Microbiology, University and Hospital Trust of Verona, Verona, Italy; ^5^Immunology Section, Department of Medicine, University and Hospital Trust of Verona, Verona, Italy; ^6^Intensive Care Unit, Department of Surgery, Dentistry, Maternity and Infant, University and Hospital Trust of Verona, Verona, Italy

**Keywords:** SARS-Cov-2, bronchial aspirate samples, mechanically ventilated patients, *Pseudomonas aeruginosa*, *Klebsiella pneumoniae*, bacterial superinfection, antimicrobial resistance

## Abstract

**Objective:**

To investigate the presence of bacteria and fungi in bronchial aspirate (BA) samples from 43 mechanically ventilated patients with severe COVID-19 disease.

**Methods:**

Detection of SARS-CoV-2 was performed using Allplex 2019-nCoV assay kits. Isolation and characterisation of bacteria and fungi were carried out in BA specimens treated with 1X dithiothreitol 1% for 30 min at room temperature, using standard culture procedures.

**Results:**

Bacterial and/or fungal superinfection was detected in 25 out of 43 mechanically ventilated patients, generally after 7 days of hospitalisation in an intensive care unit (ICU). Microbial colonisation (colony forming units (CFU) <1000 colonies/ml) in BA samples was observed in 11 out of 43 patients, whereas only 7 patients did not show any signs of bacterial or fungal growth. *Pseudomonas aeruginosa* was identified in 17 patients. Interestingly, 11 out of these 17 isolates also showed carbapenem resistance. The molecular analysis demonstrated that resistance to carbapenems was primarily related to OprD mutation or deletion. *Klebsiella pneumoniae* was the second most isolated pathogen found in 13 samples, of which 8 were carbapenemase-producer strains.

**Conclusion:**

These data demonstrate the detection of bacterial superinfection and antimicrobial resistance in severe SARS-CoV-2-infected patients and suggest that bacteria may play an important role in COVID-19 evolution. A prospective study is needed to verify the incidence of bacterial and fungal infections and their influence on the health outcomes of COVID-19 patients.

## Introduction

In the current era of multi-drug-resistant bacteria, a new viral pandemic due to the SARS-CoV-2 virus began near the end of 2019. SARS-CoV-2 is the cause of coronavirus disease 2019 (COVID-19), which had infected 113 472 187 people and resulted in 2 520 653 deaths worldwide as of 02 March 2021 (WHO website). Coronaviruses are an important viral family composed of seven viruses: four of these (HKU1CoV, OC43CoV, NL63CoV, and 229ECoV) are related to mild respiratory diseases, whereas the other three (SARS-CoV, SARS-CoV-2, and MERS-CoV) are associated with severe pneumonia. In particular, SARS-CoV induced severe acute respiratory syndrome (SARS) outbreaks in 2002 and 2003, with 10% mortality (Drosten et al., 2003; Ksiazek et al., 2003), while MERS-CoV caused severe respiratory disease outbreaks in 2012 in the Middle East, with 36% mortality (Zaki et al., 2012).

SARS-CoV-2 was first isolated in several cases of pneumonia of unknown origin in Wuhan, China; three months later, WHO declared a SARS-CoV-2 pandemic. The origin of SARS-CoV-2 is not fully understood, but the high genome sequence homology with bat and pangolin coronaviruses suggests a possible spillover from bats to humans through pangolins, with the acquisition of an essential basic amino acid sequence in the viral S protein (Letko et al., 2020). The spread of SARS-CoV-2 infection occurred quickly and the clinical impact was dramatic, especially in comorbid patients >65 years old. Notwithstanding the viral damage to pulmonary structures, SARS-CoV-2-induced alteration of the immune response has also been observed in critically ill COVID-19 patients (Pedersen and Ho, 2020; Bost et al., 2021).

In this paper, we investigated the co-occurrence of bacterial superinfections in severe COVID-19 patients. It is well known that the patients at the greatest risk for multi-drug-resistant infections are those who are already more vulnerable to viral lung infections (McCullers, 2014). This association has severe implications for global health, as bacterial co-infection and/or superinfection leads to both increased morbidity and mortality (Smith and McCullers, 2014; Morris et al., 2017). While the impacts of co-infection with bacteria and COVID-19 have not been well studied, people who are already immunocompromised by one organism are usually much more susceptible to a secondary infection and increased mortality. Interestingly, superinfection is also considered an important risk factor for COVID-19-related mortality (Mirzaei et al., 2020; Zhang et al., 2020).

Several antivirals and antimicrobials have been suggested for the management of COVID-19, with contrasting and unclear results (Morris et al., 2017; Rawson et al., 2020). Select antimicrobial therapy appears to be effective in the treatment of suspected or confirmed bacterial respiratory co-infection or superinfection; this therapy may be empiric or targeted and be conducted in patients presenting to the hospital or for the management of nosocomial infections. Recently, Cox *et al.* (Cox et al., 2020) highlighted the importance in the analysis and management of COVID-19 patients’ co-infections of determining whether these induce disease progression and appropriately tailoring antimicrobial stewardship. It is vital that we understand whether the COVID-19 pandemic may have contributed to an increase in antimicrobial resistance, since this is a growing problem with implications for both global health and the world economy (NoAuthor, 2020).

In order to pinpoint the dynamics of bacterial and/or fungal superinfection in SARS-CoV-2 patients, we investigated the presence of bacteria and fungi in bronchial aspirate (BA) samples of mechanically ventilated patients with severe COVID-19 disease.

## Materials and Methods

### Sample Population and Data Collection

We analysed BA samples obtained during routine clinical specimen collection from 43 hospitalised patients with confirmed SARS-CoV-2 infection who were subjected to mechanical ventilation (MV) between March and April 2020. BA samples were collected as part of the normal diagnostics routine from 43 patients with clinical symptoms of SARS-CoV-2 infection that required mechanical ventilation.

### Viral Infection Determination

The samples were transferred using viral SARS-Cov-2 extraction kits, and amplification was performed using Allplex 2019-nCoV assay kits (Seegene Inc., Seoul, South Korea). This multiplex real-time RT-PCR assay is based on simultaneous amplification of three viral target genes (E, N, and RdRP).

At admission, multiplex Allplex respiratory panel assays (Seegene Inc.) for the detection of 17 viruses (influenza virus A [H1, H3] and B; RSV A and B; adenovirus; enterovirus; rhinovirus; coronavirus [OC43, 229E, NL63]; bocavirus, metapneumovirus, and parainfluenza virus [PIV 1, 2, 3, 4]) were performed using nasopharyngeal swab samples.

### Bacterial Cultures

The BA samples were cultured to determine the presence of bacterial or fungal co-infections/superinfections. We considered superinfection a bacterial/fungal infection acquired after 48 h after admission of a COVID-19 patient (Garcia-Vidal et al., 2021). Samples appearing viscous were treated with 1X dithiothreitol 1% (Sigma, St Louis, USA), a reducing mucolytic agent, for 30 min at room temperature. Twenty microliters (20 µl) of treated samples were streaked out on various agar media—namely, blood agar, chocolate agar, Columbia Nalidixic acid agar, McConkey agar, mannitol salt agar, and Sabouraud dextrose agar—in order to identify all pathogens. Samples were incubated at 37°C for 18–24 h, and semi-quantitative analysis of bacterial and fungal growth was performed. Bacterial/fungal load of 10^5^–10^6^ colony forming units (CFU)/ml was considered as infection, while bacterial/fungal load lower than 10^3^ CFU/ml was considered as coloniser.

### Bacteria Identification and Antimicrobial Susceptibility Tests

All isolated strains were identified using a VITEK MS MALDI-TOF system (BioMérieux, Marcy L’Etoile, France). Antimicrobial susceptibility was determined using an agar diffusion test, and the results were interpreted following the EUCAST 2019 criteria https://eucast.org/clinical_breakpoints/.

### Resistance Tests

The presence of carbapenemase enzymes was investigated using i) the NG-Test CARBA 5 (NG-Biotech, Guipry, France), an immunochromatographic assay that uses a multiplex lateral flow immunoassay (LFIA) to detect NDM, KPC, VIM, IMP, and OXA-48-like enzymes, and ii) specific PCR to determine the presence of the *bla_KPC_* gene (Mazzariol et al., 2012), with the amplicons sequenced (MWG Operons, Ebersberg, Germany) as indicated in the subsequent sections. OprD porin that is involved in the carbapenem resistance of *P. aeruginosa* strains was also investigated. Deletions or mutations of the *oprD* gene were analysed by PCR (Ocampo-Sosa et al., 2012) and sequenced.

## Results

### Cohort Characteristics and Infection Data

We studied 43 severe COVID-19 patients. All patients were hospitalised between March and April 2020 and exhibited positive detection of viral genes through a SARS-CoV-2 RT-PCR assay. The average age of the patients was 61.4 years, and 21% of the patients were female.

### Analysis of Microbiological Samples

**Figure 1:**
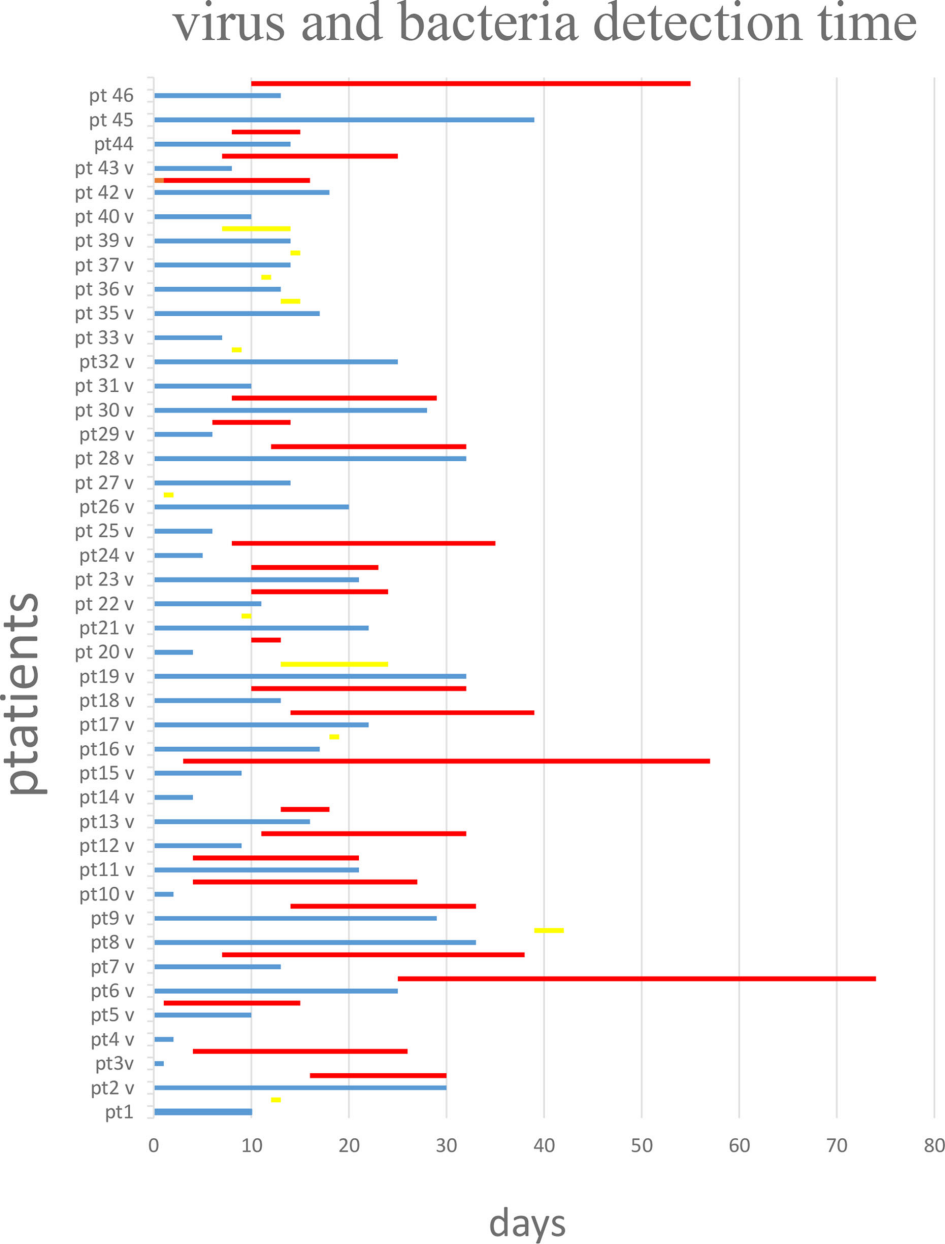
Virus (blue lines) and bacteria (red line if high load, yellow line if in low load) detection over the time starting from day 0 that represent the admission in intensive care unit. Virus and bacteria were detected in the BA of the 43 patients with COVID-19 disease. Blue line: days of virus detection. Red line: days of bacteria detection in high load. Yellow line: days of bacteria detection in low load.

**Figure 2:**
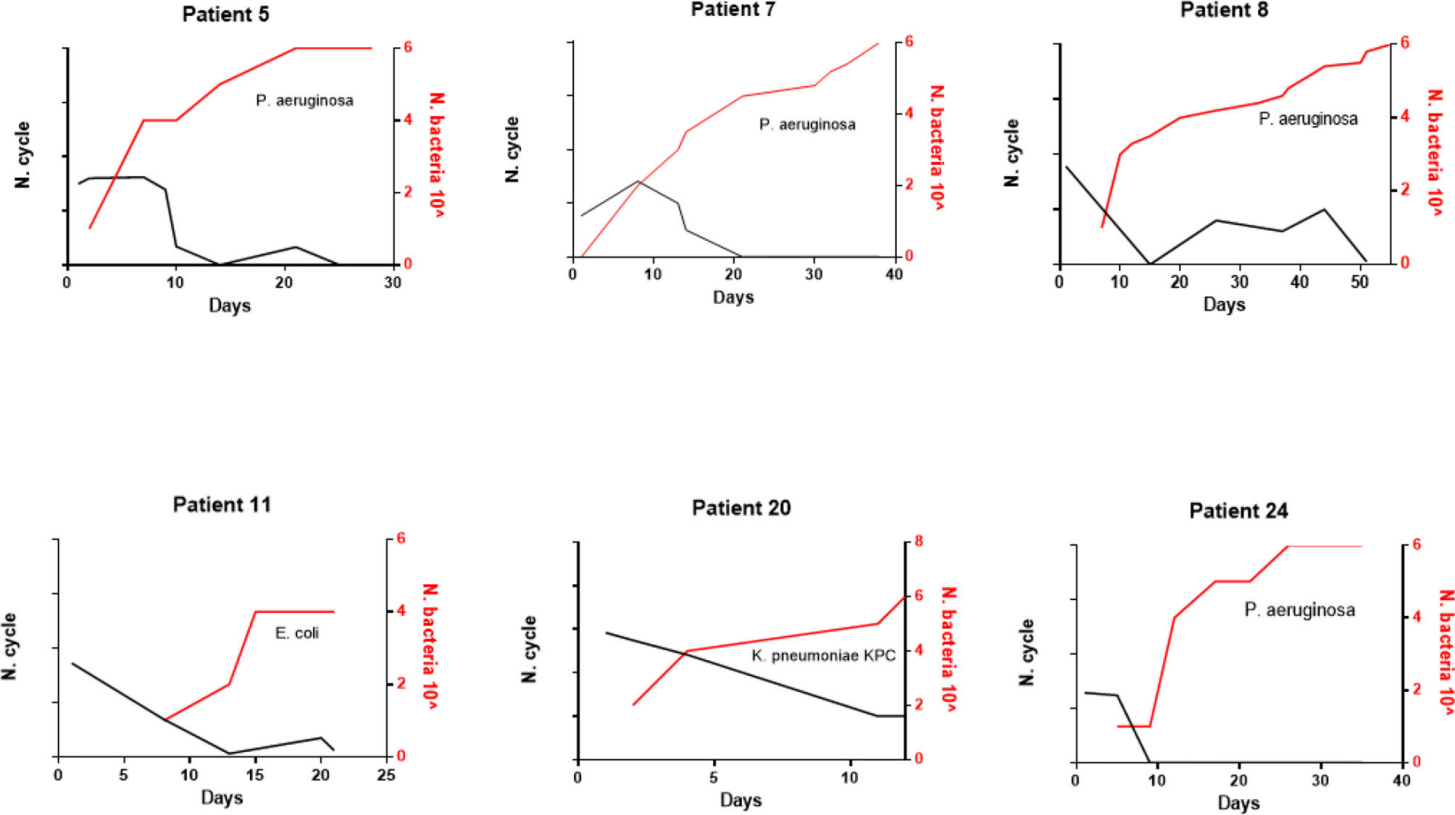
Evolution of SARS-CoV-2 c/t detection (black line) and bacteria load (red line) over the time of hospitalization in six representative patients.

All patients were followed for SARS-CoV-2 infection during the clinical course of the disease. The RT-PCR determination of three major viral target genes (E, N, and RdRP) demonstrated the presence of viral infection through BA samples. The data in **Figure 1** show when bacterial colonization/infection started after admission to the intensive care unit (ICU) and demonstrate that positive test results were obtainable from patients for a variable number of days. Bacteria were typically detected 5–15 days after hospital admission. **Figure 2** represents the relationship between the presence of SARS-CoV-2 and bacterial load. It is noteworthy that while all patients started with viral infections, the Ct value of viral gene amplification decreased in all patients until it was no longer detectable. Meanwhile, in the BA samples of these patients, increasing loads of *P. aeruginosa* and/or *K. pneumoniae* were recovered. When the bacteria reached a high load, the virus was usually no longer detectable, at least in the BA samples; the bacteria took over and constituted the major actors in the evolution of the infectious process.

Interestingly, bacterial and/or fungal superinfection was detected in 25 out of 43 patients (58.13%), generally after 7 days in the ICU with MV. Microbial colonisation (CFU <1000 colonies/ml) in BA samples was observed in 11 out of the 43 patients, whereas only seven patients did not show any signs of bacterial or fungal growth. **Table 1** reports the bacterium and fungus species isolated from BA samples, with the semi-quantitative load and the detection of SARS-CoV-2.

**Table 1:** SARS-CoV-2 and bacteria detection for each patient with superinfection are reported with the SARS-CoV-2 gene N c/t and the bacterial identification and load at the corresponding day of hospitalization.

Patient	Days of hospitalization	SARS-CoV-2 gene N c/t	Bacteria	Bacterial load
2	1	23.31		
	9	29.83		
	16	33.78		
	21		*E. aerogenes*	+++
	23	36.22	*P. aeruginosa*	+
	30	32.41	*P. aeruginosa*	+++
			*K. pneumoniae*	+
3	20	23.70	*P. aeruginosa*	+++
	26		*P. aeruginosa*	+++
4	2	29.39	*K. pneumoniae*	+
5	1	25.02	*P. aeruginosa*	+
			*K. pneumoniae*	+
	7	23.79	*P. aeruginosa*	+
	8	–	*E. faecium*	+
	9	26.07		
	15	NR	*P. aeruginosa*	++
	21	26.68		
6	1	21.91		
	12	33.50		
	14	33.36		
	18	34.40		
	25	39.31		
	26	NR	*S. maltophylia*	+++
	28	NR	*S. maltopylia*	+++
	30	NR	*S. maltophylia*	+
	32	NR	*S. maltophylia*	+++
	35	NR	*E. faecalis*	+
	46	NR	*S. maltophylia*	+
	54	NR	*S. maltophylia*	+++
	58	NR	*S. maltophylia*	+
			*K. pneumoniae*	+
	74	NR	*P. aeruginosa*	+++
7	2	32.27	*P. aeruginosa*	+
	13	25.85		
	14		*P. aeruginosa*	+
			*K. pneumoniae*	++
	21		*P. aeruginosa*	+++
			*K. pneumoniae*	+
	30		*P. aeruginosa*	+
	31		*P. aeruginosa*	++
	32		*P. aeruginosa*	+
			*C. albicans*	++
	34		*P. aeruginosa*	+
	38		*P. aeruginosa*	+++
8	2	22.10		
	10		*Yeasts*	+
	12	15.07		
	15		*Yeasts*	++
	26			
	33	26.81		
	37	25.21		
	38	33.96		
	40	34.15	*P. aeruginosa*	++
	42		*P. aeruginosa*	++
	44	28.15		
	50	38.23		
	51	39.44		
9	8	28.90		
	15	33.35	*E. aerogenes*	+
	23	39.79	*K. pneumoniae*	+++
	29	39.25	*K. pneumoniae*	+
	36	36.34		
	37		*P. aeruginosa*	++
			*K. pneumoniae*	+
			*E. aerogenes*	rco
10	1	24.54		
	5		*E. cloacae*	++
			*P. mirabilis*	+
			*Yeasts*	+
	19		*P. aeruginosa*	+
	26		*P. aeruginosa*	+
	27		*P. aeruginosa*	+++
12	1	26.71		
	2	29.08		
	5	31.06		
	8	28.88		
	16		*K. pneumoniae*	++
	22	38.53	*K. pneumoniae*	+
	27		*K. pneumoniae*	+
	29		*P. aeruginosa*	+
	31		*K. pneumoniae*	+
			*P. aeruginosa*	+
13	1	26.23	*K. pneumoniae*	+
	15	36.13	*K. pneumoniae*	+
	16	38.86		
	17		*K. pneumoniae*	+++
15	1	28.69		
	2	23.55		
	4		*E. aerogenes*	+
			*P. aeruginosa*	++
	6	36.39		
	7		*P. aeruginosa*	+++
	11		*P. aeruginosa*	+
	18		*P. aeruginosa*	+
	33	35.80	*P. aeruginosa*	+
	38		*P. aeruginosa*	++
	41		*P. aeruginosa*	+++
	45	37.91		
	46		*P. aeruginosa*	+
	57		*P. aeruginosa*	+
17	1	16.54		
	8	30.70		
	15	38.92	*P. aeruginosa*	+++
	22	38.36	*P. aeruginosa*	++
	25		*P. aeruginosa*	+
			*K. pneumoniae*	+
	38		*P. aeruginosa*	
			*K. pneumoniae*	
	39		*P. aeruginosa*	
			*K. pneumoniae*	
19	1	26.96		
	10	31.51		
	15		*P. aeruginosa*	+
	17	32.14		
	22		*P. aeruginosa*	+
	25	38.23	*P. aeruginosa*	+
	26			
	31	38.21		
	32	36.66		
20	1	20.77		
	2	22.54		
	4	25.85		
	11		*K. pneumoniae*	++
	12		*K. pneumoniae*	+++
22	1	24.40		
	11	33.13	*P. aeruginosa*	+++
	14		*P. aeruginosa*	+
	24	16.84	*P. aeruginosa*	++
24	1	27.12		
	5	27.58		
	10		*P. aeruginosa*	+
	13		*P. aeruginosa*	+
	20		*P. aeruginosa*	+
	24		*P. aeruginosa*	++
	33		*P. aeruginosa*	+++
	35		*P. aeruginosa*	+++
28	1	18.14		
	6	18.20		
	13	23.80	*K. pneumoniae*	+
			*C. albicans*	++
	14	26.92		
	18	25.62	*K. pneumoniae*	+
	25	23.65		
	32	38.11	*K. pneumoniae*	++
	34	39.12		
35	1	19.01		
	5	27.98		
	7	24.43		
	10	24		
	14	30.5	*P. aeruginosa*	+
	17	30.39	*K. pneumoniae*	+
39	1	20.33		
	6	22.33		
	8	30.06	*P. aeruginosa*	+
	11	30.11		
	14	30.19	*P. aeruginosa*	+++
	19	34.66		
	20	34.42		
42	1	25.05	*E. coli*	+++
	2	28.31		
	4			
	9	27.76	*E. coli*	+++
	12	28.98		
	15	33.63	*E. coli*	++
	18	32.88		
43	1	27.27		
	3	30.32		
	8	28.67	*P. aeruginosa*	+
	10			
	13		*P. aeruginosa*	+
	19	38.48	*P. aeruginosa*	+
	25		*P. aeruginosa*	++
46	1	24.85	*H. parainfluenzae*	+
			*Streptococcus spp*	+
	11	24.83		
	13	26.44		
	24		*P. aeruginosa*	+
	36		*P. aeruginosa*	+
	45		*P. aeruginosa*	+
	46		*P. aeruginosa*	++
	47		*P. aeruginosa*	+++

+ : 10^1^-10^2^-10^3^

+: 10^4^

++: 10^5^

+++10^6^

Analysis of the bacteria and fungi involved in these superinfections showed that the most frequently isolated bacterial species was *P. aeruginosa*, which was found in 17 out of 43 patients (39.53%), all of whom had bacterial superinfections (68% of superinfected patients). Eleven out of 17 isolates were carbapenem-resistant *P. aeruginosa*, considered to be multi-drug-resistant strains. Nine of these 11 carbapenem-resistant *P. aeruginosa* strains were detected in sequential BA samples with a high load, ranging from 10^5^ to 10^6^ CFU/ml. The remaining six (i.e., non-carbapenem-resistant) *P. aeruginosa* isolates produced antibiotic-susceptible strains. However, only one of these susceptible *P. aeruginosa* strains played a role as an infective agent; the other five isolates can be considered simply as coloniser strains. It is noteworthy that in one patient (number 7, **Table 2**), *P. aeruginosa* infection started with a susceptible strain that subsequently acquired resistance to antibiotics.

**Table 2:** Antimicrobial profile, mechanisms of resistance, and load of the two main pathogens recovered in superinfected patients.

Patient N.	*P. aeruginosa*						*K. pneumoniae*						*Outcome*
	Antimicrobial profile				Resistance mechanisms	Load	Antimicrobial profile				Resistance mechanisms	Load	
	MPM	CAZ	CIP	AK			MPM	CAZ	CIP	AK			
2	R	S	S	S	OprD MUT	***	S	R	R	S	ESBL	*	Other ICU
3	R	S	R	S	OprD DEL	***	S	R	R	S	ESBL	*	DEAD
4	–	–	–	–	–	–	S	S	S	S	–	*	Pneumology
5	S	S	S	S	–	**	S	S	S	S	–	*	Infectioous disease
6	S	S	S	S	–	***	R	R	R	S	KPC	*	Pneumology
7	S→R	S→R	S→R	S	OprD DEL	***	S	S	S	S	–	**	DEAD
9	R	S	S	S	OprD MUT	**	R	R	R	S	KPC	***	DEAD
10	R	S	S	S	OprD MUT	*	–	–	–	–	–	–	Pneumology
12	R	S	S	–	OprD MUT	*	S	I	S	S	ESBL	**	DEAD
13	–	–	–	–	–	–	R	R	R	S	KPC	***	Other ICU
15	R	R	S	S	OprD MUT	***	–	–	–	–	–	–	Pneumology
17	R	R	S	S	OprD DEL	***	R	R	R	R	KPC	*	Pneumology
19	S	S	S	S	–	*	–	–	–	–	–	–	DEAD
20	–	–	–	–	–	–	R	R	R	I	KPC	***	Other ICU
22	S	S	S	S	–	***	–	–	–	–	–	–	Pneumology
24	R	R	R	S	OprD DEL	**	–	–	–	–	–	–	Other ICU
28	–	–	–	–	–	–	R	R	R	R	KPC	***	Other ICU
35	S	S	S	S	–	***	R	R	R	S	KPC	***	Medicine
39	S	R	S	S	–	***	–	–	–	–	–	–	Pneumology
43	R	S→R	S	S	OprD DEL	**	–	–	–	–	–	–	Pneumology
46	R	R	S	S	OprD MUT	***	R	R	R	S	KPC	*	Pneumology

*: 10^1^; 10^2^; 10^3^

*: 10^4^; **: 10^5^; ***: 10^6^

MPM, Meropenem; CAZ, ceftazidime; CIP, Ciprofloxacin; AK, Amikacin; R, Resistant; S, Susceptible; OPR D MUT, mutations present in the Porin D of P. aeruginosa affecting meropenem resistance; OPR D DEL, deletion of the porin D gene in P. aeruginosa affecting meropenem resistance; ESBL, Extended Spectrum Beta-Lactamase; KPC, Klebsiella pneumoniae carbapenemase; ICU, intensive care unit.

We also investigated the characteristics of antibiotic resistance in *P. aeruginosa* strains. Resistance to carbapenems in multi-drug-resistant *P. aeruginosa* isolates was related to *oprD* mutation or deletion (**Table 2**), not the detection of carbapenemases. Five out of 11 carbapenem-resistant *P. aeruginosa* strains involved in infection showed *oprD* gene deletion and produced a negative result following *oprD*-specific PCR amplification. The other six resistant strains showed successful *oprD* gene amplification; however, after sequencing, the gene displayed insertions, deletions, or mutations with amino acid substitution, with deleterious consequences for OprD expression and synthesis. As expected, all six susceptible strains presented an *oprD* gene encoding for a functional porin that facilitates carbapenem entry into the cell.

As reported in **Table 2**, the second most isolated pathogen was *K. pneumoniae*, detected in samples from 13 out of 43 patients (30.23%) and representing 52% of the superinfected patients (13 out 25). The phenotypic distribution of the 13 K*. pneumoniae* strains consisted of two susceptible strains, three ESBL-producer strains, and eight carbapenemase-producer strains. All the carbapenemase-producer strains harboured a *bla_KPC_* gene and expression was confirmed by CARBA 5 immunochromatographic tests. Both ESBL and carbapenemase producers were multi-drug resistant, with few therapeutic choices. In addition, 8 out of the 13 (61.53%) *K. pneumoniae* isolates exhibited high loads and were isolated in multiple BA samples from these patients, while 6 were KPC producers.

Nine patients were infected with both *P. aeruginosa* and *K. pneumoniae*; in four of these individuals, high loads of both strains were detected. In these high-load patients, the *P. aeruginosa* strains always showed a resistant pattern, while the *K. pneumoniae* strains exhibited variable patterns of susceptibility—namely, one susceptible, one ESBL producer, and two KPC producers. Other bacteria that were isolated and play a role as an infectious agent were *Stenotrophomonas maltophilia* and *Escherichia coli*, which were each detected only in one case. High loads of fungi were detected in just one patient, together with *E. coli* superinfection; otherwise, fungi were found only as colonisers at very low loads (less than 10^3^ CFU/ml).

## Discussion

The SARS-CoV-2 virus is the etiological agent of COVID-19. This airborne infection can present as a SARS with the need for MV. WHO reported mortality rates ranging from less than 0.1% to over 25% in different countries, while reports from Johns Hopkins University estimated a mortality rate of 3–4% for COVID-19 patients. Pedersen *et al.* (Pedersen and Ho, 2020) reported studies showing increased levels of IL-6, IL-10 and TNF-α in SARS-CoV-2-infected individuals. The concentrations of these inflammatory cytokines, which are also involved in other systemic inflammatory syndromes such as cytokine storms, declined during patients’ recovery from SARS-CoV-2 infection (Liu and Hill, 2020; Liu et al., 2021). Interestingly, bacterial superinfection plays a very important role in influenza virus infection and other respiratory virus infections. A review by McCullers (Bost et al., 2021) reported the complex interaction between bacterial and influenza viruses in pulmonary pathogenesis, indicating that bacterial superinfections might promote severe disease and mortality.

Cox et al. (2020) underlined the importance of the accurate management of co-infections and the evaluation of related antimicrobial resistance. To elucidate this aspect, we collected microbiologic data from 43 severe COVID-19 patients. Diagnosis was performed using real-time PCR, and all patients showed SARS-CoV-2 target amplification at early Ct values. All patients were admitted to the ICU and required MV. BA samples were analysed for SARS-CoV-2, and some were also cultured to investigate the presence of bacteria and fungi. Over half (58.13%) of patients showed the presence of high loads of bacteria. These data might indicate an important role of bacterial secondary infection. The occurrence of bacterial co-infections/superinfection reported in COVID-19 patients ranges from 5.1% (Chen et al., 2020) to 29.8% (Zhang et al., 2020). Interestingly, as shown in **Figure 2**, SARS-CoV-2 Ct values always progressively declined until becoming undetectable, while bacterial load simultaneously increased.

*P. aeruginosa*, which was isolated from 68% of patients showing bacterial infection, played a very important role in superinfection. High loads of carbapenem-resistant *P. aeruginosa* were detected in 10 out of 11 patients. These data are intriguing as they increase our knowledge about SARS-CoV-2 bacterial superinfections. Indeed, Mirzaei *et al.* (Mirzaei et al., 2020) reported the known microorganisms that co-infected and/or were responsible for pneumonia in SARS-CoV-2-infected patients. They listed atypical pneumonia agents such as *Mycoplasma pneumoniae* and *Legionella pneumophila* that were not found in our patients, along with bacteria such as *Staphylococcus aureus*, *K. pneumoniae*, *Enterobacter cloacae*, and *Acinetobacter baumannii*. This discrepancy between *Mirzaei et al.*’s findings and our study could be related to differences in epidemiology. The second most frequently detected microorganism was *K. pneumoniae*, another classic pathogen responsible for nosocomial pneumonia. In this case as well, multi-drug-resistant microorganisms were predominant. Eight out of 13 strains were KPC-producer strains that are endemic in Italy (Giani et al., 2013). Six patients exhibited high loads of these strains.

Both multi-drug-resistant *P. aeruginosa* and *K. pneumoniae* showed carbapenem resistance, although this was supported by different mechanisms. Our investigation showed that KPC production by *K. pneumoniae* and *oprD* gene deletion or mutation in the case of *P. aeruginosa* results in a non-functional outer membrane channel are responsible of carbapenem resistance. These data support the idea that we are faced with hypermutable strains of *P. aeruginosa*. This hypothesis must be investigated further, together with the possible genetic relatedness of isolates.

This work analysed the impact of SARS-CoV-2 infection on antimicrobial resistance. Concerns about antimicrobial resistance did not disappear during the pandemic; rather, it has become a pivotal concern in the management of SARS-CoV-2-infected patients. The use of antibiotics to treat pneumonia caused by COVID-19 must take into account the fact superinfection could, in many cases, be sustained by multi-drug-resistant microorganisms with a substantial impact on therapeutic choices. This is even more important when patients are admitted to the ICU, a ward where antimicrobial resistance can develop and spread easily, especially if there is normally a high incidence of resistance. In our study, we documented a 58.13% incidence of superinfection in SARS-CoV-2-infected patients admitted to the ICU. Most of the patients in our study were infected with carbapenem-resistant *P. aeruginosa*, followed by multi-drug-resistant *K. pneumoniae*.

Rhee et al. (2020) recently reported clinical evidence suggesting increased mortality related to inadequate empiric antibiotic treatment. In this scenario, the microbiology laboratory would play a pivotal role both in identifying bacterial superinfections and determining resistance patterns and the underlying mechanisms of resistance, so as to enable health care providers to make the appropriate choices concerning antimicrobial treatments.

## Conclusions

These data suggest that bacteria may play an important role in COVID-19 evolution and that antimicrobial resistance might negatively impact outcomes for affected patients. A prospective study will be necessary to verify the incidence of bacterial and fungal infections and their influence on COVID-19 outcomes.

## Data Availability Statement

The original contributions presented in the study are included in the article/supplementary material. Further inquiries can be directed to the corresponding author.

## Author Contributions

AM and DG: conceptualization. IU, RN, and MS: investigation. AM, ABen, and IU: formal analysis. AM and ABen: data curation. ABen, FDS, SU, KD, and EP: resource. AM, ABen, and DG: writing original draft. AM, FS, SU, KD, EP, and DG: writing review and editing. All authors contributed to the article and approved the submitted version.

## Funding

This study was supported by the Cariverona Foundation, ENACT project VIRO-COVID.

## Conflict of Interest

The authors declare that the research was conducted in the absence of any commercial or financial relationships that could be construed as a potential conflict of interest.

## Publisher’s Note

All claims expressed in this article are solely those of the authors and do not necessarily represent those of their affiliated organizations, or those of the publisher, the editors and the reviewers. Any product that may be evaluated in this article, or claim that may be made by its manufacturer, is not guaranteed or endorsed by the publisher.
